# Effects of a 6-week semi-supervised exercise training program on physical fitness and mental health in post-COVID-19 healthcare workers: a randomized clinical trial

**DOI:** 10.1186/s13102-025-01317-w

**Published:** 2025-09-03

**Authors:** Feng Gao, Fei Liu, Yongfu Liu, Zhi Li, Wenjun Yu, Yuxin Xiao, Di Liu, Shuaikang Yang, Song Yuan, Junhua Wang, Shenglong Le

**Affiliations:** 1 Department of Physical Therapy, Taihe Hospital, Hubei University of Medicine, Shiyan, China; 2 Center for Diabetes Rehabilitation Research, Taihe Hospital, Hubei University of Medicine, Shiyan, China; 3 School of Biomedical Engineering, Hubei University of Medicine, Shiyan, China; 4 Department of Rehabilitation, Taihe Hospital, Hubei University of Medicine, Shiyan, China; 5 Department of Physical Education, Shanghai Jiao Tong University, Shanghai, China

**Keywords:** post-COVID-19, Exercise therapy, Muscle function, Health personnel, Physical fitness

## Abstract

**Background:**

Healthcare workers rank among the groups that are most vulnerable to the SARS-CoV-2 infection. Deterioration in physical fitness and mental health is a common manifestation in the post-COVID-19 person, which require specific and effective treatment. This study aimed to evaluate the effects of a 6-week, semi-supervised exercise intervention on physical fitness and mental health in post-COVID-19 healthcare workers (HCWs).

**Methods:**

Fifty-four HCWs infected with SARS-CoV-2 within 3–4 weeks were randomly assigned to an exercise group or a control group. The exercise group followed a 6-week semi-supervised program with three weekly 60–80-minute sessions. Assessments included handgrip strength, sit-to-stand, push-up, curl-up, cardiorespiratory fitness, flexibility, balance, and mental health. Analyses followed intention-to-treat principles.

**Results:**

Compared with those of the control group, the push-up scores (*p* = 0.003, η_p_^2^ = 0.162), curl-up scores (*p* < 0.001, η_p_^2^ = 0.248), and maximum load of the rowing ergometer (*p* = 0.012, η_p_^2^ = 0.121) significantly increased in the exercise group. The depression (*p* = 0.003) and anxiety (*p* = 0.023) scores significantly decreased at the end of the intervention in the exercise group, although no significant differences were observed between the groups. No adverse events potentially linked to the intervention were observed.

**Conclusions:**

A six-week semi-supervised exercise training significantly improved functional muscle strength and endurance, and maximum rowing intensity in post-COVID-19 HCWs compared to controls, along with within-group reductions in depression and anxiety. No adverse events were reported. These findings suggest that exercise may be a safe and effective recovery strategy to prevent or alleviate post-COVID-19 conditions, although further research is needed to confirm long-term effectiveness and mechanisms of action.

**Trial registration:**

The study was registered in the Chinese Clinical Trial Registry (#ChiCTR2100051901) from 2021-10-09.

## Introduction

The coronavirus disease 2019 (COVID-19) pandemic, induced by severe acute respiratory syndrome coronavirus 2 (SARS-CoV-2), has profoundly disrupted daily life worldwide. As of March 2023, more than 676 million individuals had been infected with SARS-CoV-2, with 6.8 million succumbing to the disease [1]. Approximately 10–12% of infected people, including those who were already vaccinated, experienced ongoing symptoms after the acute phase of the disease [2]. This condition is correlated with considerable health dysfunctions, such as impaired cardiorespiratory performance, intolerance to physical exertion, decreased muscle mass and psychological dysfunctions (e.g., anxiety and depression) [2, 3]. Accumulating evidence suggests that these post-acute symptoms resulting from SARS-CoV-2 infection may arise from complex underlying pathophysiological mechanisms, including inflammation and immune dysregulation, endothelial dysfunction, mitochondrial dysfunction, and autonomic nervous system alterations [4–8]. Collectively, these mechanisms underscore the need for integrative approaches to its management.

Healthcare workers (HCWs), such as physicians, nurses, and healthcare assistants, who are continuously exposed to the risk of SARS-CoV-2 infection, are the most susceptible to SARS-CoV-2 infection, with a hazard ratio of 11.6 against the general population [9]. More than half of HCWs have reported post-COVID-19 conditions [10, 11]. With regard to the physically demanding and emotionally draining tasks that HCWs perform, this is a major concern [12]. Persistent symptoms can also have adverse effects on the healthcare delivery system, resulting in a potential loss of proficient HCWs as a consequence of the continuous increase in their work absences and the continuous decline in their productivity [13].

Rehabilitation based on exercise has shown potential as a treatment approach for those who have recovered from COVID-19, but its impacts rely on symptoms [14]. Recent evidence indicates that rehabilitation with physical exercise as the main component has positive effects on people who have suffered from SARS-CoV-2 infection, alleviating symptoms and addressing both acute and post-acute consequences of the disease [14–18]. Among emerging interventions, inspiratory muscle training is increasingly recognized for its potential to improve exercise tolerance, particularly in the post-COVID-19 patients with residual dyspnea or fatigue [19–21]. Despite these developments, further research is still needed. The majority of current studies have focused primarily on patients who have been hospitalized or persons with long-term COVID-19 commonly diagnosed by a healthcare provider at least three months after SARS-CoV-2 infection [22]. Additionally, little research has been carried out among HCWs. A study on the post-COVID-19 recovery of surviving HCWs who were working at a large hospital in Vienna has been reported, but it lacked a control group [23]. New concerns have also emerged regarding the effectiveness and safety of physical activity shortly after recovery from COVID-19 [24]. Particularly in low- and middle-income countries, HCWs are often required to return to frontline duties shortly after testing negative, without sufficient recovery or rehabilitation support. In light of these clinical uncertainties, there is a critical need for studies that examine the efficacy and safety of structured physical activity interventions, especially in high-risk groups such as HCWs.

Thus, this study aimed to evaluate the efficacy and safety of 6 weeks of semi-supervised exercise training on the physical fitness and mental health of post-COVID-19 HCWs through a randomized clinical trial. This contributes to the growing field of post-COVID-19 rehabilitation by focusing on a critically important but understudied population, offering evidence to inform targeted interventions that can support HCWs in safely and effectively returning to work following COVID-19.

## Methods

### Study design and setting

This blinded assessor randomized controlled trial (RCT) was conducted between December 26, 2022, and March 30, 2023, in a tertiary public hospital in Shiyan, China. The study was approved by the Institutional Review Board of the researchers’ institution and conducted in accordance with the Declaration of Helsinki. All participants provided written informed consent. This report adhered to the Consolidated Standards of Reporting Trials (CONSORT) reporting guidelines for RCTs [25].

### Participants

Potential participants were recruited from December 26, 2022, to January 8, 2023, via brochures and social media campaigns targeting HCWs. The inclusion criteria were (1) aged between 18 and 70 years; (2) aged between 3 and 4 weeks after SARS-CoV-2 infection; and (3) ability to exercise. Candidates were excluded if they (1) had positive reverse-transcription‒polymerase chain reaction (RT‒PCR) SARSCoV-2 test results from a nasopharyngeal or oropharyngeal swab; (2) had a history of any contraindications for exercise, including but not limited to uncontrolled diabetes, severe musculoskeletal disorders preventing physical activity, unstable angina, significant arrhythmia, and respiratory conditions with severe functional limitation; (3) had a resting heart rate above 100 beats per minute; (4) had a blood pressure below 90/60 mmHg or above 140/90 mmHg or blood pressure fluctuation over 20 mmHg, accompanied by obvious symptoms of discomfort; or (5) had a blood oxygen saturation ≤ 95%.

**Fig. 1:**
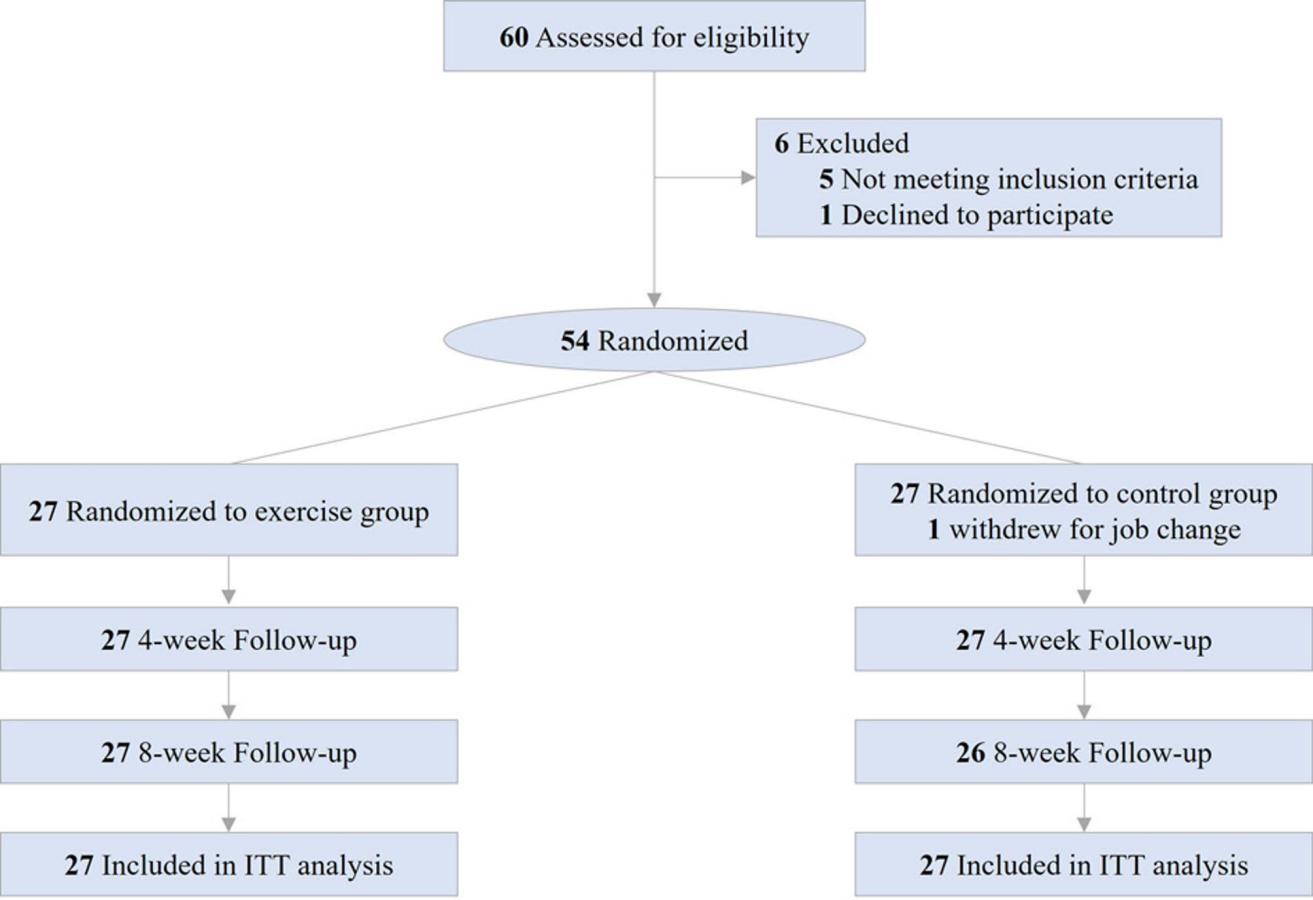
Study flow diagram. ITT, intention-to-treat

Fifty-four participants were randomly assigned at a 1:1 ratio to the exercise (Exe) or control (Con) group before the first measurement through an automated computer-generated randomization protocol administered by the principal investigator not involved in the measurement (Fig. 1). Blinding of participants was not feasible. However, researchers involved in outcome assessments remained blinded to treatment allocation until the completion of the study.

### Exercise protocol

**Fig. 2:**
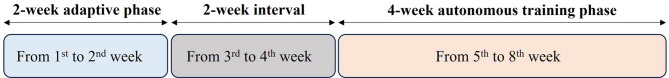
Graphical illustration of the 6-week, semi-supervised, progressive exercise intervention

The participants in the Exe group underwent a 6-week, two-phase, semi-supervised, progressive exercise intervention 3 times per week, totaling 18 sessions (Fig. 2). Each session lasted 60–80 min as follows: warm-up (5–10 min), endurance training (20 min), strength training (30–40 min) and cool-down (5–10 min). During the first 2-week phase, physiotherapists taught and supervised participants to perform exercise to ensure proper performance and safety, followed by the second 4-week phase without supervision. Owing to the Chinese New Year holiday, there was a 2-week interval between the two phases.

The strength training included movements targeting the legs, arms, chest, back, and abdomen, such as lunges, heel raises, abdominal crunches, and chest expansion. During the first phase, each movement was performed in two sets (12–15 repetitions) with 60–90 s of rest under the instruction of physiotherapists. During the second phase, each movement was performed in 3 sets (20–30 repetitions) with 60–90 s of rest.

The endurance training was conducted on a rowing ergometer. The load was 3 for the female and 7 for the male during the first phase, and a 50–60% maximal load for both genders was measured at the 5th week during the second phase. Heart rate was monitored by a Polar H10 heart rate monitor (Polar Electro Oy, Kempele, Finland).

The participants in the Con group were instructed to maintain their regular activities.

### Outcomes

#### Primary outcomes

The primary outcomes were functional muscle strength and endurance, which were assessed via handgrip strength measurements and 60-second sit-to-stand tests, push-up tests, and curl-up tests, following protocols from the Chinese National Physical Fitness Monitoring Manual. A handheld dynamometer (EH101, Zhongshan Camry Electronic Co., Ltd., China) was employed to assess the handgrip strength of the dominant hand. In the 60-second sit-to-stand test, the subjects were required to sit on a standard chair with a backrest and a height of 40–45 centimeters and then stand up from the chair and sit back down as many times as possible within 60 s.

The primary outcomes were measured at the beginning and end of the entire intervention.

#### Secondary outcomes

The secondary outcomes included anthropometry, body composition, cardiorespiratory fitness, flexibility and balance capacity, and mental health. A bioimpedance device (InBody 770, Biospace, Co., Ltd., Seoul, Korea) was used to assess body mass and body composition. The sit-reach test and one-leg standing test with the eyes closed with the dominant leg were used to assess flexibility and balance capacity, respectively, according to the Chinese National Physical Fitness Monitoring Manual.

The submaximal step test (Step Index) was used to assess cardiovascular fitness according to the Chinese National Physical Fitness Monitoring Manual. The maximal graded exercise test was also carried out on an ergometer (Monark 839E, Monark, Vansbro, Sweden). After a 3-minute 50-watt warm-up, the workload was increased by 30 watts every 3 min until exhaustion, despite verbal encouragement. Peak oxygen consumption (VO_2peak_) was measured breath-by-breath through a computerized system (K5, COSMED, Rome, Italy). The maximal load test of the rowing ergometer (Load_max_) was assessed by increasing the load by 1 level of loading every 2 min until exhaustion, despite verbal encouragement.

For the assessment of mental health, the Self-Rating Depression Scale (SDS) and the Self-Rating Anxiety Scale (SAS) were utilized to assess the degree of depression and anxiety, respectively [26, 27]. A positive relationship existed between the scores obtained from the scale and the level of depression or anxiety.

The secondary outcomes were measured at the beginning and end of the entire intervention, except for VO_2peak_ and Load_max,_ which were assessed at the beginning and end of the second training phase.

#### Adverse events

Throughout the study, participants were at liberty to report any adverse events at any juncture, by phone call or WeChat message, or by verbally informing the therapist directly. The participants were encouraged to report any adverse events during training and assessments.

### Sample size

Given the limited knowledge about the effects of exercise training on muscle strength and endurance in post-COVID-19 patients, a previous study on the effects of a 6-week supervised exercise training program on musculoskeletal performance (push-up) in patients recovering from severe acute respiratory syndrome was used in the sample size calculation [28]. Using 80% statistical power, a 5% significance level (α = 0.05), and a 10% estimated dropout rate, 26 subjects per group were needed. This value was calculated via the ClinCalc statistical calculator.

### Statistical analysis

All outcomes were analyzed via intention‒to‒treat (ITT) principles, following CONSORT guidelines. The regression imputation method was used to process the missing VO_2peak_ and Load_max data_, with age, sex, and the step index as independent variables. Continuous variables are presented as the means ± standard deviations (SDs), whereas categorical variables are presented as frequencies and percentages. The distribution normality was examined via the Shapiro‒Wilk normality test. Differences between groups at baseline were assessed by the chi-square test, an independent sample t test or the Mann‒Whitney U test. Intragroup differences between pre- and postintervention values were compared through paired t tests. The effects of exercise training on the outcomes were assessed via repeated measures analysis of variance (ANOVA), with age, sex, and baseline values used as covariates. Effect size was quantified via partial eta squared (η_p_^²^), with thresholds defined as small (0.01), medium (0.06), and large (0.14) [29]. The significance level was set at *p* < 0.05. All the statistical analyses were performed via IBM SPSS Statistics for Windows (version 21.0) and RStudio for Windows (version 2024.12.0).

## Results

### Participant characteristics

A total of 54 HCWs (age, 31.6 ± 6.7 years; women, 25 (46.3%); all Chinese) were randomly assigned to the Exe group (*n* = 27) or the Con group (*n* = 27) and included in the ITT analysis. One participant withdrew from the study at week 6 due to job transfer; therefore, 53 participants completed the follow-up assessments (Fig. 1). Table 1 presents a summary of the demographic and clinical characteristics of the participants at baseline. No statistically significant differences were observed between the two groups at baseline.

**Table 1:** Participant characteristics at baseline

Characteristic	Exercise group (*n* = 27)	Control group (*n* = 27)
Age, mean (SD), y	32.7 (7.7)	30.6 (5.5)
Gender, No. (%)		
Female	13 (48.1)	12 (44.4)
Male	14 (51.9)	15 (55.6)
Marriage status, No. (%)		
Unmarried	11 (40.7)	9 (33.3)
Married	16 (59.3)	18 (66.7)
Weight, mean (SD), kg	64.2 (11.3)	64.4 (13.1)
Body mass index, mean (SD)^a^	23.2 (2.5)	23.0 (3.1)
Medical history, No. (%)		
Current smoker	1 (3.7)	3 (11.1)
Alcohol	10 (37.0)	9 (33.3)
Diabetes	1 (3.7)	0 (0)
Hyperlipidemia	1 (3.7)	1 (3.7)
Educational level		
Associate’s degree	0 (0)	1 (3.7)
Bachelor’s degree	18 (66.7)	23 (85.2)
Master’s degree	9 (33.3)	3 (11.1)
Symptoms of COVID-19, No. (%)		
Fever	27 (100)	25 (92.6)
Myalgia	24 (88.9)	23 (85.2)
Loss/weaken of smell/taste	18 (66.7)	17 (63.0)
Nasal congestion/runny nose	23 (85.2)	21 (77.8)
Sore throat	24 (88.9)	21 (77.8)
Cough	25 (92.6)	23 (85.2)
Nausea/vomiting	10 (37.0)	10 (37.0)
Taking any medication, No. (%)	25 (92.6)	20 (74.1)
PA levels, mean (SD), METs/week ^b^	25.8 (21.5)	14.9 (19.4)

SD, standard deviation; COVID-19, coronavirus disease 2019; PA, physical activity; MET: metabolic equivalent

^a^ Calculated as weight in kilograms divided by height in meters squared

^b^ Physical activity level before SARS-CoV-2 infection assessed by the questionnaire

### Primary outcome

**Fig. 3:**
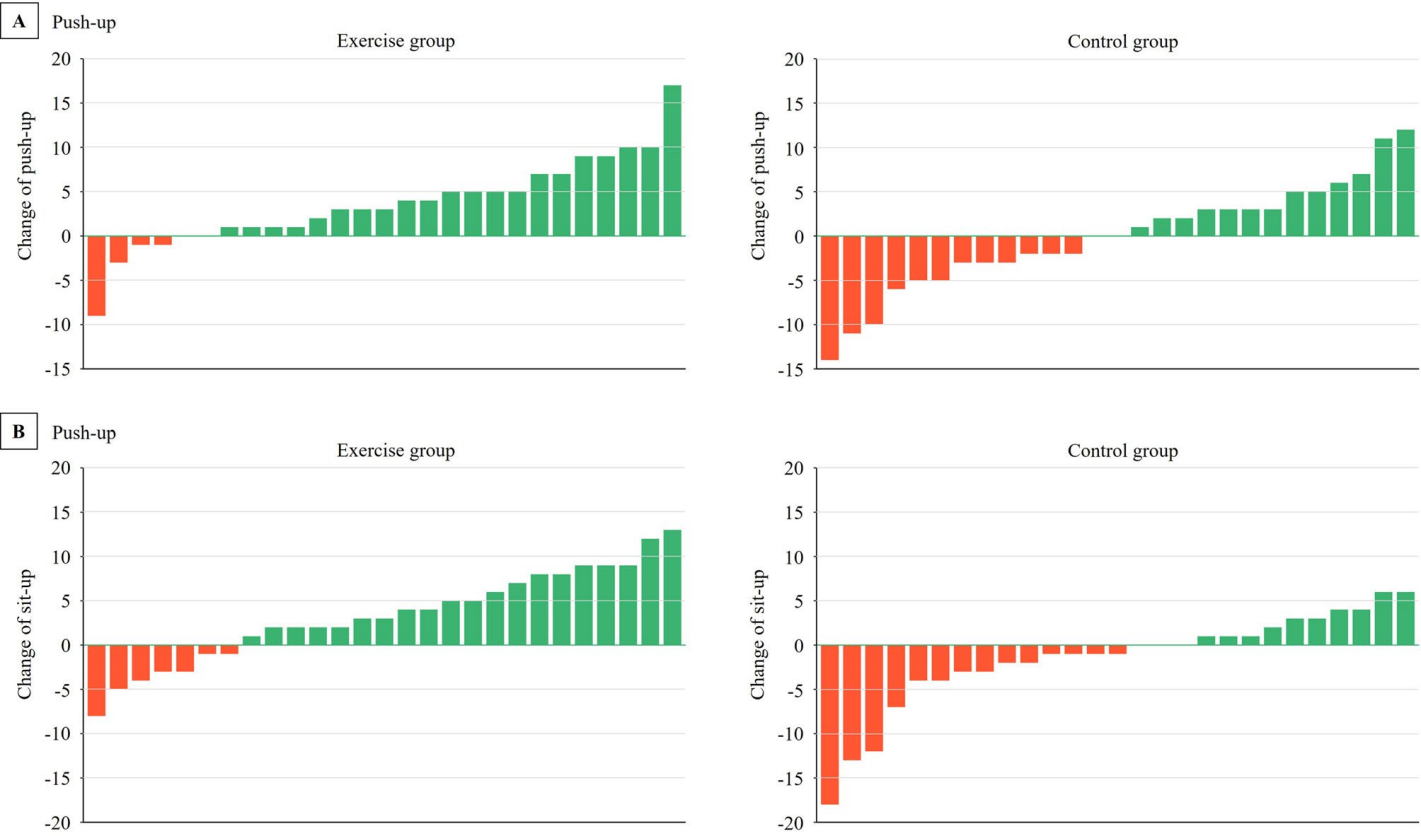
Individual changes in push-up and curl-up after the 6-week intervention

Push-up (*p* = 0.001), curl-up (*p* = 0.003), and 60-second sit-to-stand (*p* = 0.025) were significantly improved by the end of the intervention in the Exe group (Table 2). After adjustment for age, sex and baseline values via ANOVA, the push-up (*p* = 0.003, η_p_^2^ = 0.162) and curl-up (*p* < 0.001, η_p_^2^ = 0.248) significantly improved in the Exe group compared with the Con group (Table 2; Fig. 3). No significant differences between the groups were found in handgrip strength or 60-second sit-to-stand time after the intervention (*p* > 0.05) (Table 2).

**Table 2:** Posttest results and ANCOVA showing the effects of 6-week semisupervised supervised exercise training (exercise) compared with no intervention (control) in post-COVID-19 healthcare workers

Variable	Control			Exercise			Group effect ^a^	
	Pre	Post		Pre	Post		*p*	η_p_^2^
Body composition								
Body mass (kg)	64.4 (13.1)	65.2 (13.6) ^*^		64.2 (11.3)	65.2 (11.4) ^*^		0.594	0.006
Fat mass (%)	26.9 (5.5)	26.9 (6.3)		26.3 (6.0)	26.9 (5.5)		0.461	0.011
Lean mass (%)	68.9 (5.3)	68.9 (6.0)		69.4 (5.8)	68.9 (5.3)		0.539	0.008
Muscle strength and endurance								
Handgrip (kg)	34.8 (12.5)	35.7 (12.8)		34.8 (11.0)	35.7 (11.4)		0.871	0.001
Push-up	18.2 (8.2)	18.0 (7.7)		21.9 (12.3)	25.5 (13.3) ^*^		**0.003**	**0.162**
Sit-up	19.5 (8.4)	18.0 (6.6)		19.6 (8.2)	22.9 (7.4) ^*^		**0.000**	**0.248**
60-second sit-to-stand	43.5 (7.6)	45.9 (9.8)		46.1 (12.0)	51.2 (12.2 ^*^		0.062	0.070
Flexibility and balance capacity								
Sit-reach (cm)	8.6 (6.4)	8.5 (8.2)		10.6 (7.7)	10.3 (6.8)		0.929	0.000
One-leg standing (s)	26.4 (18.5)	33.7 (27.1) ^*^		22.2 (20.6)	44 (36.8) ^*^		0.075	0.063
Cardiovascular fitness								
Load_max_^b^	11.5 (2.3)	11.2 (2. 2)		12.3 (2.6)	12.8 (2.8)		**0.012**	**0.121**
Step Index^c^	113.2 (30.8)	113.1 (23.2)		113.2 (29.3)	115.9 (18.4)		0.840	0.001
VO_2peak_ (ml/kg/min) ^d^	28.6 (5.6)	28.8 (5.9)		30.8 (5.1)	32.5 (8.2)		0.284	0.023
Mental health								
SDS	48.0 (12.1)	43.7 (14.5)		54.2 (8.1)	46.3 (14.1) ^*^		0.929	0.000
SAS	38.5 (7.9)	36.3 (9.5)		44.1 (8.5)	40.4 (10.3) ^*^		0.614	0.005

Note: Data are shown as the means and standard deviations (SDs)

η_p_^2^, partial eta squared effect size; SDS, Self-Rating Depression Scale; SDS, Self-Rating Anxiety Scale

^a^ Repeated-measures analysis of variance with age, sex, and baseline values as covariates

^b^ Maximal load of the rowing ergometer

^c^ Estimated by the submaximal step test according to the Chinese National Physical Fitness Monitoring Manual

^d^ Assessed by the maximal graded exercise test carried out on the cycling ergometer

^*^ *p* < 0.05, paired t test

### Secondary outcome

After adjustment for age, sex and baseline values via ANOVA, the Load_max_ of the rowing ergometer (*p* = 0.012, η_p_^2^ = 0.121) significantly improved within the Exe group compared with the Con group (Table 2; Fig. 3). Both the Exe group (*p* = 0.005) and the Con group (*p* = 0.009) showed an increase in body mass by the end of the intervention (Table 2). Additionally, both groups demonstrated significant improvements in balance capacity (Exe group: *p* = 0.002; Con group: *p* = 0.046). No significant differences were found between the groups in terms of body composition, flexibility, balance capacity, step index, or VO_2peak_ after the intervention (*p* > 0.05) (Table 2).

With respect to mental health parameters, the SDS scores (*p* = 0.003) and SAS scores (*p* = 0.023) were significantly lower in the Exe group at the end of the intervention (Table 2). However, after adjusting for age, sex, and baseline values via ANOVA, no significant improvements in SDS or SAS scores were observed in the Exe group compared with the Con group (*p* > 0.05) (Table 2).

### Adherence and adverse events

The participants in the Exe group completed 4.0 ± 0.2 of the 6 scheduled training sessions during the 2-week adaptive training phase (adherence rate: 67.3%). During the subsequent 4-week training phase (unsupervised), they completed 11.2 ± 1.0 of the 12 scheduled sessions (adherence rate: 93.5%).

No adverse events potentially associated with the intervention were observed during the study.

## Discussion

To the best of our knowledge, this is one of the first RCTs investigating the effectiveness of exercise interventions in post-COVID-19 HCWs who continue working on the front line. Our results demonstrated that a 6-week semi-supervised rehabilitation program consisting of multicomponent exercises significantly improved functional muscular strength and endurance, as evidenced by enhanced performance in the push-up and curl-up tests, with a large effect size.

This study cohort comprises post-COVID-19 HCWs, who, owing to continuous exposure to SARS-CoV-2, face a greater risk of reinfection and shorter intervals between infections [30, 31]. Moreover, more than half of the participants experienced post-COVID-19 symptoms, such as fatigue and muscle weakness. These symptoms pose a significant challenge given the physically and emotionally demanding nature of healthcare work [10–12]. Considering the high workload of HCWs and limited access to rehabilitation resources, this study highlights the importance of developing effective and accessible training modalities that can help HCWs recover from post-COVID-19 fatigue and musculoskeletal impairment without complications or adverse effects.

Following the 6-week exercise intervention, measurable improvements in muscular performance were observed. Proximal muscle strength and endurance improved, as reflected in enhanced push-up and curl-up performance. These improvements are likely attributable to the structured exercise program. In contrast, for lower body strength, although there were significant within-group changes from pre- to postintervention in the intervention group, these changes were not statistically significant compared with those in the control group. This finding is partially inconsistent with those of previous studies. For example, Sick et al. reported that 12 weeks of supervised endurance training or concurrent training improved lower body strength in individuals who were infected at least 12 weeks prior to enrollment [17]. Similarly, Del Corral et al. demonstrated that 8 weeks of aerobic exercise training, with or without respiratory muscle training, improved 60-second sit-to-stand performance among those with long COVID-19 [15]. Hasenoehrl et al. reported that an 8-week program combining supervised resistance training twice a week with self-directed aerobic exercise thrice a week improved 30-second sit-to-stand performance in HCWs with post-COVID-19 symptoms [23]. The discrepancy may be due to the shorter duration of the current intervention (only 6 weeks), although there was still a trend toward intergroup improvements (*p* = 0.062). Consistent with the results of previous studies, we did not observe significant changes in handgrip strength either within or between groups [17, 32, 33].

Given the negative impact of SARS-CoV-2 on cardiorespiratory function, most previous rehabilitation studies have focused on improving cardiorespiratory fitness [15, 17, 23]. These interventions typically involve aerobic exercise over an 8–12-week period and increase VO_2peak_ in long-term COVID-19 patients [15, 17, 23]. However, our findings differ in this regard. While our intervention improved the maximal load on the rowing ergometer, there were no significant improvements in VO2peak measured on the cycling ergometer or in the submaximal step test. This difference may be due to the specificity of training. Unlike previous studies that included longer durations of cycling, walking, or running, our program included only 20 min of endurance training on the rowing ergometer, which focused primarily on functional muscle strength and endurance [15, 17, 23].

Psychiatric symptoms such as anxiety and depression are frequently reported following recovery from acute SARS-CoV-2 infection [34]. In the present study, however, there was no significant improvement in anxiety or depression scores between the intervention and control groups. This contrasts with the findings of Hasenoehrl et al., who reported that an 8-week exercise intervention significantly reduced depression and anxiety in post-COVID-19 HCWs [23]. The difference may be attributed to the study design. Hasenoehrl et al. did not include a non-exercise control group, whereas our study did include one. Notably, in both the exercise group and the control group, anxiety and depression scores decreased significantly compared with those at baseline, which aligns with the findings of Hasenoehrl et al. This finding suggests that these symptoms may naturally diminish over time or be influenced by other factors.

Reduced muscle strength and endurance are frequent long-term consequences observed in individuals who have recovered from even mild forms of COVID-19 [2, 3, 7]. Several underlying mechanisms may contribute to this muscle impairment, including inflammation, mitochondrial dysfunction, endothelial dysfunction, and autonomic dysregulation [4–8]. Chronic systemic inflammation, characterized by elevated pro-inflammatory cytokines, which impair vascular function and skeletal muscle oxygen delivery [8, 35]. Concurrent microvascular injury following SARS-CoV-2 infection may further degrade circulatory efficiency, especially during physical exertion [36]. Mitochondrial dysfunction, often due to disrupted oxidative phosphorylation, contributes to persistent fatigue and decreased ATP production [5, 37]. Similarly, disturbances in autonomic nervous system regulation—such as impaired heart rate recovery and postural tachycardia—may exacerbate subjective fatigue and psychological distress [4].

Against this backdrop, a six-week semi-supervised resistance- and rowing-based exercise program significantly improved muscle strength and endurance in HCWs recovering from COVID-19. These improvements can be attributed to several mechanisms inherent to structured physical activity. First, resistance training promotes muscle hypertrophy and contractile efficiency through increased protein synthesis and mTOR activation, counteracting the catabolic effects of chronic inflammation [38]. Second, resistance training also enhances mitochondrial health, a key target in post-COVID-19 rehabilitation. It can stimulate mitochondrial biogenesis via PGC-1α activation, improving ATP production and antioxidant capacity [39, 40]. Third, resistance exercise can positively influence autonomic nervous system function [41, 42]. Lastly, the semi-supervised model may also have improved adherence and motivation, which are crucial for rehabilitation success. While these mechanisms are consistent with current evidence, it is important to note that further research is needed to validate these pathways in post-COVID-19 patients.

### Strengths and limitations

This study makes a significant contribution by providing one of the first controlled evaluations of a semi-supervised multicomponent exercise program for post-COVID-19 healthcare workers. Our findings demonstrate that even a relatively short intervention of 6 weeks can lead to meaningful improvements in upper body muscular strength and endurance, suggesting the potential of low-resource, accessible interventions in this population. The semi-supervised model is particularly relevant in occupational settings where direct supervision is not always feasible. While the intervention showed promising outcomes for proximal muscle function, it was less effective in improving lower body strength and psychological symptoms, which may highlight the need for extended or more comprehensive rehabilitation models. These results, therefore, contribute to the growing evidence supporting structured physical activity as a safe and effective approach for post-COVID-19 recovery, especially in high-risk professional groups.

However, the current study has several limitations. First, the generalizability of the findings may be limited, as HCWs often have a higher level of health awareness and better stress management abilities than the general population does. Additionally, most participants were able to return to work and engage in the intervention program despite recent infections. The study results may not apply to other groups, such as adolescents or older adults, who may experience more severe post-COVID-19 symptoms or be more affected by the disease. Second, the intervention period of 6 weeks may be too short to yield significant between-group differences for all outcomes. Extending the program to 8–12 weeks could enhance the observable benefits. Third, without validated baseline physical performance data or comparisons with pre-infection values, it is difficult to distinguish between physical deconditioning caused by SARS-CoV-2 and preexisting occupational fatigue. Despite this, the majority of HCWs reported experiencing post-COVID-19 symptoms such as fatigue and muscle weakness, and over half of our participants reported a negative impact of the infection on their physical health, work, and daily life [10–12]. Therefore, our study remains highly relevant for the management and prevention of post-COVID-19 complications in HCWs.

## Conclusions

This study demonstrated that a six-week semi-supervised exercise training significantly improved functional muscle strength and endurance, and maximum rowing intensity in post-COVID-19 HCWs compared to controls, along with within-group reductions in depression and anxiety. No adverse events were reported. These findings suggest that exercise may be a safe and effective recovery strategy to prevent or alleviate post-COVID-19 conditions, although further research is needed to confirm long-term effectiveness and mechanisms of action.

## Data Availability

All data of this study are available from the corresponding author upon reasonable request.

## Abbreviations

ANOVA: Analysis of variance
COVID-19: Coronavirus disease 2019
HCWs: Healthcare workers
ITT: Intention-to-treat
η_p_²: Partial eta squared
RCT: Randomized controlled trial
SAS: Self-Rating Anxiety Scale
SDS: Self-Rating Depression Scale
SD: Standard deviation
VO_2peak_: Peak oxygen consumption
